# An infant with Cushing syndrome

**DOI:** 10.1186/1687-9856-2015-S1-P41

**Published:** 2015-04-28

**Authors:** Andrew Sng, Kah Yin Loke, Yvonne Lim, Cindy Ho

**Affiliations:** 1 National University Hospital, Singapore

## Background

Infantile Cushing syndrome is rare. We report an 8 month-old infant who developed growth failure and hypertension from an apparently innocuous topical steroid application.

## Case Presentation

An 8 month-old boy was referred to the endocrinology clinic for concerns of growth failure since 3 months of age. He was born full term with a birth weight of 2.67kg and a length of 45cm. His parents noticed that he was not growing well from 3 months of age. However, his weight gain remained more than satisfactory and he gained 700g in the past month. This infant was otherwise in good health, apart from atopic dermatitis, for which the parents had been applying a daily cream with good effect. He was breast fed until 2 months of age and weaned onto solids at 6 months of age.

On examination, the infant had a moon face with hypertrichosis, facial telangiectasia and prominent supraclavicular fat pads. His recumbent length was 62cm (<3^rd^ percentile), his weight was 7.74 kg (25^th^ percentile), and his head circumference was 41.0 cm (3^rd^ percentile). There was no hyperpigmentation or lentigenes, but he was hypertensive with a blood pressure of 140/90 mmHg (95^th^ percentile for age is 99/55 mm Hg) with a heart rate of 140 beats/minute. His apex beat was not displaced and his heart sounds were normal with no murmurs.

A random steroid profile demonstrated a depressed serum cortisol level of 30 nmol/L and an unmeasurably low serum ACTH level of <1.1 pmol/L. This was consistent with exogenous Cushing syndrome. An ECG showed biventricular hypertrophy and a 2-D echocardiogram confirmed severe biventricular hypertrophy with good ventricular function.

On further questioning, he had been receiving twice daily applications of a topical cream for atopic dermatitis, which had been obtained over the counter, without a prescription. Subsequent analysis of the topical agent revealed the contents to be betametasone dipropionate, a high potency corticosteroid.

On establishing the diagnosis of exogenous Cushing syndrome, the offending topical steroid cream was discontinued. Since he was at risk of primary adrenal failure from sudden withdrawal of steroid cream, he was started on a weaning regimen of hydrocortisone. He was also commenced on captopril which achieved good blood pressure control.

## Conclusion

Continuous use of high potency topical corticosteroids over several months can lead to Cushing syndrome. Physiologic dosing of hydrocortisone should be prescribed to prevent an adrenal crisis until hypothalamic-pituitary-adrenal axis recovery is confirmed.

Written informed consent was obtained from the patient for publication of this abstract and any accompanying images. A copy of the written consent is available for review by the Editor of this journal.

